# “The War on Ageism” or How Ageism Is Reduced During Wartime

**DOI:** 10.1093/geroni/igaf122.915

**Published:** 2025-12-31

**Authors:** Liat Ayalon, Sarit Okun, Assaf Suberry

**Affiliations:** Bar-Ilan University, Ramat-Gan, HaMerkaz, Israel; Bar-Ilan University, Ramat-Gan, HaMerkaz, Israel; Bar-Ilan University, Ramat-Gan, HaMerkaz, Israel

## Abstract

**Objectives:**

The study evaluated the perceptions of lay people in Israel concerning the presence of ageism during the Swards of Iron war.

**Methods:**

An online survey was administered to 911 Israelis during the month of April 2024. Respondents provided responses to two open-ended questions concerning their general views on ageism during the war in general as well as their own personal experiences with ageism during the war.

**Results:**

In total, 50% of the responses discussed a reduction or non-existence of ageism during the war in general, whereas 72% of the responses discussed such a reduction or non-existence of ageism based on their own personal experience during the war. This was attributed to: a) growing collaboration between young and old, b) younger people’s willingness to sacrifice their lives, and c) older persons’ contribution during the war.

**Discussion:**

The findings are discussed in the context of the contact theory and the theory of successful aging. It is of note that a horrific situation such as the war and neo-liberal values that advocate for activity and contribution may have unexpected positive consequences in the form of reduced ageism.

